# Visualization of epicardial lead infection using ^18^F‐FDG‐PET/CT imaging

**DOI:** 10.1002/joa3.12515

**Published:** 2021-02-13

**Authors:** Tasuku Kurokawa, Takanori Arimoto, Naoaki Hashimoto, Daisuke Kutsuzawa, Shigehiko Kato, Tetsu Watanabe, Ken Watanabe, Daisuke Ishigaki, Tetsuro Uchida, Masafumi Watanabe

**Affiliations:** ^1^ Department of Cardiology, Pulmonology, and Nephrology Yamagata University Faculty of Medicine Yamagata Japan; ^2^ Second Department of Surgery Yamagata University Faculty of Medicine Yamagata Japan

**Keywords:** ^18^F‐FDG‐PET/CT, epicardial lead infection

## Abstract

^18^ F‐FDG‐PET/CT is promising tool to visualize not only transvenous lead infection but also epicardial lead infection.

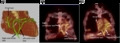

## 1 CARDIAC ARRHYTHMIA SPOT LIGHT

**FIGURE 1:**
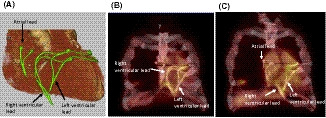
A, Three‐dimensional computed tomographic image of the whole heart. Atrial, right ventricular, and left ventricular leads were implanted on the epicardium. B,C, Coronal views of ^18^F‐FDG‐PET showed local increase in ^18^F‐FDG accumulation in the atrial, right ventricular, and left ventricular leads. Maximum standardized uptake value was 2.5 in the atrial lead, 3.6 in the right ventricular lead, and 5.0 in the left ventricular lead, respectively

Positron emission tomography/computed tomography (PET/CT) with glucose analogue 2‐[18F] fluoro‐2‐deoxy‐D‐glucose (^18^F‐FDG) can help in the detection of transvenous lead infection, but its role in epicardial lead infection is still uncertain. A 79‐year‐old man underwent coronary artery bypass graft, mitral valve repair, tricuspid annuloplasty, and epicardial cardiac resynchronization therapy (CRT) pacemaker implantation 7 years ago. Two years later, generator had been removed due to CRT pocket infection. Because of his frailty, complete device removal was abandoned; and after initial treatment with antibiotics, a watchful waiting policy was adopted. Fortunately, his hemodynamics status had been stable without CRT support after generator removal. This time, he was admitted for bradyarrhythmia with atrial fibrillation and complete atrioventricular block. There was a small fistula near the incision and local cultures showed growth of methicillin‐resistant *Staphylococcus caprae*. There were no signs such as localized erythema and pain in the pocket area. A whole‐body ^18^F‐FDG‐PET/CT scan (Biograph mCT, Siemens Healthineers, Erlangen, Germany) was performed. The patient fasted 18 hours before the PET/CT to reduce physiological glucose uptake of the myocardium. Injected dose of ^18^F‐FDG was 160 MBq. Sixty minutes later, a whole‐body PET acquisition (2 min per bed position) was performed following CT scan for anatomical reference and attenuation correction. Maximum standardized uptake value (SUV_max_) was 2.5 in the atrial lead, 3.6 in the right ventricular lead, and 5.0 in the left ventricular lead, respectively (Figure 1). The difference of SUV _max_ may reflect heterogeneous infectious conditions in each leads. There was no local increase in ^18^F‐FDG accumulation around the generator removal area. Although ^18^F‐FDG‐PET/CT clearly visualized the epicardial leads infection, performing a rethoracotomy to remove the epicardial leads was deemed as highly invasive and lethal procedure in the present case. He had no fever and had mild inflammatory response during hospitalization. In addition, two blood culture sets were negative. Therefore, our heart team decided to retain the epicardial leads and implant the Micra leadless pacemaker (Medtronic, Minneapolis, MN, USA) to maintain activities of daily living. Its intracardiac location and small surface area provide an advantage in the patient at risk of recurrent infection. The patient has been well during a 6‐month‐follow‐up period. ^18^F‐FDG‐PET/CT may be promising tool to visualize not only transvenous lead infection but also epicardial lead infection.

## CONFLICT OF INTEREST

The authors have no conflict of interest to disclose.

